# Passive electrolocation in terrestrial arthropods: Theoretical modelling of location detection

**DOI:** 10.1016/j.jtbi.2022.111357

**Published:** 2023-02-07

**Authors:** Ryan A. Palmer, Isaac V. Chenchiah, Daniel Robert

**Affiliations:** a School of Biological Sciences, University of Bristol, Life Sciences Building, 24 Tyndall Avenue, Bristol, BS8 1TQ, United Kingdom; b School of Mathematics, University of Bristol, Fry Building, Woodland Road, Bristol, BS8 1UG, United Kingdom

**Keywords:** Electroreception, Location detection, Filiform hairs, Mechanosensory, Arthropods

## Abstract

The recent discovery that some terrestrial arthropods can detect, use, and learn from weak electrical fields adds a new dimension to our understanding of how organisms explore and interact with their environments. For bees and spiders, the filiform mechanosensory systems enable this novel sensory modality by carrying electric charge and deflecting in response to electrical fields. This mode of information acquisition opens avenues for previously unrealised sensory dynamics and capabilities. In this paper, we study one such potential: the possibility for an arthropod to locate electrically charged objects.

We begin by illustrating how electrostatic interactions between hairs and surrounding electrical fields enable the process of location detection. After which we examine three scenarios: (1) the determination of the location and magnitude of multiple point charges through a single observation, (2) the learning of electrical and mechanical sensor properties and the characteristics of an electrical field through several observations, (3) the possibility that an observer can infer their location and orientation in a fixed and known electrical field (akin to “stellar navigation”).

To conclude, we discuss the potential of electroreception to endow an animal with thus far unappreciated sensory capabilities, such as the mapping of electrical environments. Electroreception by terrestrial arthropods offers a renewed understanding of the sensory processes carried out by filiform hairs, adding to aero-acoustic sensing and opening up the possibility of new emergent collective dynamics and information acquisition by distributed hair sensors.

## Highlights

• Shows unique sensory capabilities that electroreception enables.
• A single hair array can discern the location and magnitude of multiple charges.
• Several observations enable learning of an observer’s electrical field properties.
• An observer can find their location and orientation within a known electrical field.
• Shows how arthropods could map their electrical environment in real-time.

## 1 Introduction

Arthropods are ubiquitous throughout the natural world and present in nearly all ecosystems. Yet, given the vast disparities of these environments, there are remarkable common adaptions and evolutionary traits across Arthropoda, especially regarding sensing and information acquisition.

Of interest and relevance here are the passive mechanisms by which terrestrial arthropods detect and localise a signal. In passive detection, an observer derives energy from the environment for the sensing process. As discussed in Liu et al. (2022) there are many different examples of such passive signal localisation with terrestrial arthropods shown capable of pinpointing signal sources such as sound (Robert, 2005), light (Warrant and Nilsson, 2006), vibrations (Cocroft and Rodríguez, 2005, Hill and Wessel, 2016) and aero-acoustics (Casas and Dangles, 2010) through diverse sensory mechanisms.

Several of these processes depend on filiform hairs, a universal sensory adaptation of arthropods. When mechanically deflected by a stimulus, these hairs transduce information about the surrounding environment by activating one or several mechanosensory neurones at their base. Such responses to aerodynamic and acoustic stimuli have been widely investigated and are therefore well-characterised (Casas and Dangles, 2010, Kumagai et al., 1998, Tautz and Markl, 1978, Tautz, 1979, Shimozawa et al., 1998, Bathellier et al., 2005, Humphrey and Barth, 2007, Cummins et al., 2007, Koh and Robert, 2020, Barth and Höller, 1999, Steinmann and Casas, 2017, Casas et al., 2008). Recent evidence also shows that filiform hairs undergo similar deflections when exposed to weak electrical fields (for example, those of bumblebees (Sutton et al., 2016)). Furthermore, electrical interactions between an arthropod and atmospheric electrical fields have been revealed in the context of dispersal by ballooning in spiders (Morley and Robert, 2018). These ground-breaking discoveries are instructive in developing a basis for understanding the mechanisms of electroreception (the ability to detect electric fields). Studies of electroreception to date have focused on specific arthropods (e.g., spiders (Morley and Robert, 2018), bees (Clarke et al., 2017), flies (Khan et al., 2021)) and the time-independent mechanics of single (Koh and Robert, 2020, Palmer et al., 2021) or small groups of hairs (Palmer et al., 2022).

Intriguingly, like other sensory modalities, the problem of source localisation via electroreception requires consideration. Previous work shows the parametric, biological and physical feasibility of electroreception using hairs (Palmer et al., 2021), as well as the sensory mechanics of sensing electrical fields (Palmer et al., 2022). We now consider the potential use of electroreception for source localisation.

Here we introduce the notion that the electroreceptive sense is distinct since the receiver, its sensors, and its environment are all passive sources of electrical information. Indeed, electroreception is only possible due to the electrical properties of and interactions between an animal and its environment. An illustration of such interactions is how positively charged bees find and visit negatively charged flowers during pollination (Clarke et al., 2013, Clarke et al., 2017).

The remainder of this paper is organised as follows. In Section 2, we briefly present the theory of electrical sensing in arrays of N charged hairs and the equations used to model such electrical interactions. In Section 3.1 we expand our previous work (Palmer et al., 2022) to conceptually show the feasibility of location detection through hair arrays. We then present four novel sensory capabilities owing to electroreception via charged sensory hairs: detection of point charges through a single array (Section 3), learning of electrical fields and properties through observations (Section 4) and navigation via fixed and known electrical fields (Section 5). We conclude the paper with a discussion in Section 6 and conclusions in Section 7 by highlighting the power of electroreception for the above tasks, noting the limitations of the analysis, and commenting on the next steps and future questions.

## 2 Electroreception via electromechanical hairs.

### Electrical fields.

Natural electrical fields arise from several sources (e.g., global atmospheric gradients, floral electric fields) and interactions (e.g., inductive polarisation in materials, triboelectric charging) within the environment. To represent these contributions, we decompose the electrical field into three components relating to: an arthropod covered by H charged hairs each of charge $q_{h}$ located at $r_{h}$ (h=1,2,…,H), P external point charges $q_{p}$ located at $r_{p}$ (p=1,2,…,P), and some additional background component. The P point charges are considered to be predators, prey, or a distributed electrical field (e.g., due to a flower) discretised into several points. The strength and direction of the electrical field are mathematically denoted by E(r) at each point r within a given domain and written as:

(2.1) $$E\left(r\right)=\frac{1}{k_{e}}\overset{H}{\underset{i=1}{\sum }}\frac{q_{i}\left(r-r_{i}\right)}{\|r-r_{i}{\|}^{3}}+\frac{1}{k_{e}}\overset{P}{\underset{p=1}{\sum }}\frac{q_{p}\left(r-r_{p}\right)}{\|r-r_{p}{\|}^{3}}+\overset{\sim }{E}\left(r\right),$$

where $k_{e}\approx 8.988\times 10^{9}\;\left[{\mathrm{Nm}}^{2}C^{-2}\right]$ is the Coulomb constant and $\overset{\sim }{E}$ represents the background component which we take to be 0 throughout the paper (and hence neglect).

### Mechanosensory hairs as harmonic oscillators.

We now consider a single arthropod hair, say the hth hair, and denote its total charge magnitude as $q_{h}$. For simplicity, we assume all the charge is at the hair tip located at $r_{h}$. When placed within the electrical field (2.1), the hair will experience a force, $F_{h}\left(r_{h}\right)$, due to its interaction with the electrical field, given by:

(2.2) $$F_{h}\left(r_{h}\right)=q_{h}E\left(r_{h}\right).$$

**Fig. 1:**
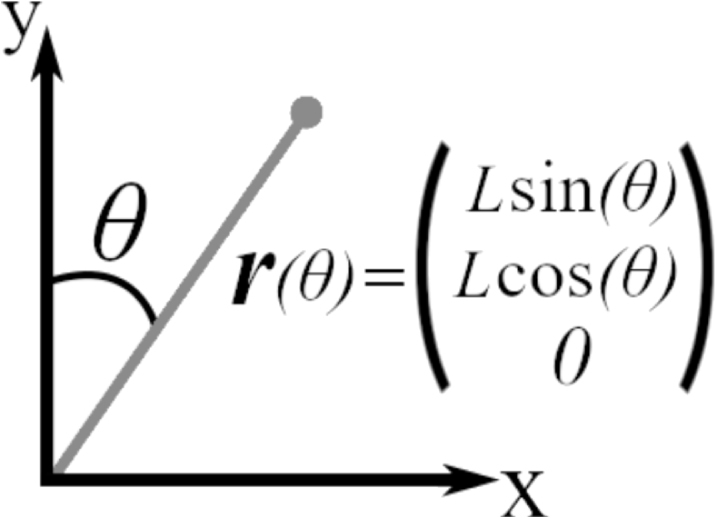
Definition of the hair co-ordinate system.

When a force is applied along the hair at a position r[m] (shown in Fig. 1), a torque $\tau =r\times F\;\left[{\mathrm{kgm}}^{2}s^{-2}\right]$ occurs that deflects the hair about the point where it attaches to the surface.

Mechanosensory hairs are modelled as rigid rods with motions described as inverted linear pendulums (Palmer et al., 2021, Kumagai et al., 1998, Shimozawa et al., 1998, Humphrey and Barth, 2007). Therefore, several features influence a hair’s motion, such as its length, shape, and the properties of the viscoelastic socket membrane (Thurm, 1965a, Thurm, 1965b). Treating the hair in this way enables us to relate the torque on the hair to the hair’s motion through

(2.3) $$-\tau =I\overset{¨}{\theta }\left(t\right)+R\overset{˙}{\theta }\left(t\right)+S\theta \left(t\right),$$

where τ=|τ| and θ[rad] is the angular deflection of the hair (i.e., the angle between the hair’s perturbed position and its resting position), $S\;\left[{\mathrm{kgm}}^{2}s^{-2}\right]$ the spring constant, $R\;\left[{\mathrm{kgm}}^{2}s^{-1}\right]$ the damping constant, and $I\;\left[{\mathrm{kgm}}^{2}\right]$ the moment of inertia (Humphrey and Barth, 2007). The dots here indicate time derivatives.

Finally, we constrain the motion of the hair to a plane (here, the x–y plane) as is common within the aerodynamic literature, e.g. Cummins et al., 2007, Humphrey and Barth, 2007. Hence, we only consider $F=\left(F_{x},F_{y},0\right)$.

As stated in the introduction, the deflection of hairs to stimuli is of biological and sensory significance since this action conveys information about an environment to an arthropod. Hence, throughout this paper, we are interested in the sensory capabilities that combinations of deflections from several hairs enable.

In the proceeding section, we detail the mathematical framework used to model arrays of sensory hairs. This method was developed in Palmer et al. (2022) to evaluate how the interactions between hairs affected their sensory capacity and collective dynamics. Here, we use the foundational model to analyse electrical localisation.

### Equations of motion.

Further considering the hth hair, h=1,2,…,H, on an arthropod. The resting position of the hair tip $r_{h}$ is defined by the location of its base, $x_{h,0}=\left(x_{h,0},y_{h,0},z_{h,0}\right)\;\left[m\right]$, its resting angle, $\theta_{h,0}\;\left[\mathrm{rad}\right]$ (measured clockwise from the y-axis), and its length, $L_{h}\;\left[m\right]$. The hairs and their motion are co-planar such that $z_{h,0}=0$.

If the hair is deflected by some angle $\theta_{h}\;\left[\mathrm{rad}\right]$, its location is given by:

(2.4) $$\left(x_{h},y_{h},z_{h}\right)=x_{h,0}+r_{h}=\left(x_{h,0},\;y_{h,0},\;0\right)+\left(L_{h}\sin\theta_{h},\;L_{h}\cos\theta_{h},\;0\right).$$

Hence, following from (2.3), the motion of a hair deflected by a force due to an arbitrary electrical field is given by:

(2.5) $$I_{h}{\overset{¨}{\theta }}_{h}+R_{h}{\overset{˙}{\theta }}_{h}+S_{h}\theta_{h}=L_{h}\cos\left(\theta_{h}\right)F_{h,x}-L_{h}\sin\left(\theta_{h}\right)F_{h,y}.$$

In this work we consider the quasi-static system such that the terms $I\overset{¨}{\theta }\left(t\right)$ and $R\overset{˙}{\theta }\left(t\right)$ are negligible (for the same reasons provided in Palmer et al. (2022)).

From the definition of the electrical field in (2.1), there are P fixed external point charges, located at $\left(x_{p},y_{p},0\right)\;\left[m\right]$, and of magnitude $q_{p}\;\left[C\right]$ ($z_{p}=0$ so that the point charges and hairs are co-planar).

The motion of the hth hair depends on the Coulomb interaction with the P point charges and the other N−1 hairs that also move in response to the external point charges and each other. Therefore, (2.1), (2.2), (2.5) give the angular deflection of the hth hair as:

(2.6) $$\theta_{h}=\frac{q_{h}}{k_{e}}\frac{L_{h}}{S_{h}}\left(\overset{P}{\underset{p=1}{\sum }}q_{p}\frac{\left(x_{h}-x_{p}\right)\cos\left(\theta_{h}\right)-\left(y_{h}-y_{p}\right)\sin\left(\theta_{h}\right)}{{\left({\left(x_{h}-x_{p}\right)}^{2}+{\left(y_{h}-y_{p}\right)}^{2}\right)}^{3/2}}\right)$$

$$\;\;+\left(\underset{i\neq h}{\sum }q_{i}\frac{\left(x_{h}-x_{i}\right)\cos\left(\theta_{h}\right)-\left(y_{h}-y_{i}\right)\sin\left(\theta_{h}\right)}{{\left({\left(x_{h}-x_{i}\right)}^{2}+{\left(y_{h}-y_{i}\right)}^{2}\right)}^{3/2}}\right).$$

To aid the analysis of (2.7) we non-dimensionalise the system to reduce the number of parameters. We, therefore, scale the equations according to

(2.7) $$\left(L_{h},S_{h},x_{h},y_{h},q_{h},x_{p},y_{p},q_{p}\right)=\left(L{\overset{\sim }{L}}_{h},S{\overset{\sim }{S}}_{h},L{\overset{\sim }{x}}_{h},L{\overset{\sim }{y}}_{h},q{\overset{\sim }{q}}_{h},L{\overset{\sim }{x}}_{p},L{\overset{\sim }{y}}_{p},q{\overset{\sim }{q}}_{p}\right)$$

where the tilde notation denotes the non-dimensionalised values. Here, L is the typical hair length, S is the typical spring constant, and q is the typical hair charge. It is expected that ${\overset{\sim }{L}}_{i},{\overset{\sim }{S}}_{i},{\overset{\sim }{q}}_{i}$ remain of order unity for i=1,2,…,N,j, whilst ${\overset{\sim }{q}}_{p}$, ${\overset{\sim }{x}}_{p}$ and ${\overset{\sim }{y}}_{p}$ may vary by several orders of magnitude. Thus, (2.7) becomes:

(2.8) $$\theta_{h}=K\frac{{\overset{\sim }{q}}_{h}{\overset{\sim }{L}}_{h}}{{\overset{\sim }{S}}_{h}}\left(\overset{P}{\underset{p=1}{\sum }}{\overset{\sim }{q}}_{p}\frac{\left({\overset{\sim }{x}}_{h}-{\overset{\sim }{x}}_{p}\right)\cos\left(\theta_{h}\right)-\left({\overset{\sim }{y}}_{h}-{\overset{\sim }{y}}_{p}\right)\sin\left(\theta_{h}\right)}{{\left({\left({\overset{\sim }{x}}_{h}-{\overset{\sim }{x}}_{p}\right)}^{2}+{\left({\overset{\sim }{y}}_{h}-{\overset{\sim }{y}}_{p}\right)}^{2}\right)}^{3/2}}\right)$$

$$\;\;+\;\left(\underset{i\neq h}{\sum }{\overset{\sim }{q}}_{i}\frac{\left({\overset{\sim }{x}}_{h}-{\overset{\sim }{x}}_{i}\right)\cos\left(\theta_{h}\right)-\left({\overset{\sim }{y}}_{h}-{\overset{\sim }{y}}_{i}\right)\sin\left(\theta_{h}\right)}{{\left({\left({\overset{\sim }{x}}_{h}-{\overset{\sim }{x}}_{i}\right)}^{2}+{\left({\overset{\sim }{y}}_{h}-{\overset{\sim }{y}}_{i}\right)}^{2}\right)}^{3/2}}\right).$$

with,

$$K=\frac{q^{2}}{k_{e}}\frac{1}{LS},$$

which is the ratio of the electrical parameters of the hair system to its mechanical parameters, which we denote as the parameter of electromechanical sensitivity. Hence, three parameters govern the hair motion: $K,{\overset{\sim }{q}}_{p}$ and the distance between the hair bases, denoted δ (Palmer et al., 2022).

### 2.1 Numerical method

Several examples of location detection and charge determination are developed throughout this article, illustrating the sensory capability of multi-hair mechanosensor systems. We solved each non-linear system using MATLAB (version 2020b). There are two choices of solver, “*fsolve*” (a constraint-free, non-linear equation solver) and “*lsqnonlin*” (a least squares method non-linear equation solver with user selected bounds). For the analyses in Section 3.1.2 and 3.2, we used both methods and found that they had the same level of accuracy and, in most cases, gave identical answers. Throughout the remainder of this article, we use the *lsqnonlin* solver since it provides more control over the solution when the scenarios become complex. Other methods for solving the equations may be more accurate or efficient; however, the purpose here is to show the capability and nuance of location detection using arrays of hairs and not to assess the best numerical methods.

Unless stated otherwise, we centre the hair arrays around the origin with the hair lengths, spring constants and charges the same for each hair ($L_{h}=S_{h}=q_{h}=1$ for h=1,2,…,12). Furthermore, we consider K=1 and with hairs spaced 0.1 hair lengths apart. These parameter choices are physically feasible and biologically relevant and lead to significant electrical coupling between hairs (as discussed in Palmer et al. (2022)).

## 3 Determining an external electrical field with known hair parameters

For a sufficient number of hairs, it is possible to determine the location and strengths of P point charges, if $L_{h}$, $q_{h}$, $\left(x_{h,0},y_{h,0}\right)$, $S_{h}$, $\theta_{h}$, and $r_{h}=\left(x_{h},y_{h},0\right)$, h=1,2,…,H, are known. To do so, the equations of motions (2.8) can be rewritten to form a solvable nonlinear system of equations. For the hth hair the equations become:

(3.1) $$A^{\left(h\right)}=\overset{P}{\underset{p=1}{\sum }}q_{p}\frac{\left({\overset{\sim }{x}}_{h}-{\overset{\sim }{x}}_{p}\right)\cos\left(\theta_{h}\right)-\left({\overset{\sim }{y}}_{h}-{\overset{\sim }{y}}_{p}\right)\sin\left(\theta_{h}\right)}{{\left({\left({\overset{\sim }{x}}_{h}-{\overset{\sim }{x}}_{p}\right)}^{2}+{\left({\overset{\sim }{y}}_{h}-{\overset{\sim }{y}}_{p}\right)}^{2}\right)}^{3/2}},$$

where

$$A^{\left(h\right)}≔\frac{{\overset{\sim }{S}}_{h}}{K{\overset{\sim }{q}}_{h}{\overset{\sim }{L}}_{h}}\theta_{h}-\overset{H}{\underset{i\neq h}{\sum }}q_{i}\frac{\left({\overset{\sim }{x}}_{h}-{\overset{\sim }{x}}_{i}\right)\cos\left(\theta_{h}\right)-\left({\overset{\sim }{y}}_{h}-{\overset{\sim }{y}}_{i}\right)\sin\left(\theta_{h}\right)}{{\left({\left({\overset{\sim }{x}}_{h}-{\overset{\sim }{x}}_{i}\right)}^{2}+{\left({\overset{\sim }{y}}_{h}-{\overset{\sim }{y}}_{i}\right)}^{2}\right)}^{3/2}},$$

is a known term.

Overall, there are 3P unknowns, $q_{p}^{\left(h\right)},x_{p}^{\left(h\right)},y_{p}^{\left(h\right)}$, p=1,2,…,P. Hence, a system of 3P polynomial equations of the form (3.1) must be solved to give the location and charge of each point charge indicating that at least 3P hairs are required to solve the system with a single observation. We explore this scenario in the proceeding section. Notably, with fewer hairs an arthropod could also make more observations (at different locations) to find a solution. This case is explored later in the paper.

The tilde notation is dropped for the remainder of the paper and all variables are henceforth non-dimensionalised.

### 3.1 Detection of a single point charge with several hairs

#### 3.1.1 Theoretical illustration

Consider an electrical field generated by a single external point charge of unknown location and magnitude. For a system of three hairs, the point charge simultaneously deflects each hair to some angle $\theta_{h},\;h=1,\;2,\;3$ as given by Eq. (2.8). Solely from the deflections and parameters of each hair, it is possible to deduce the location and magnitude of the charge as explained below.

**Fig. 2:**
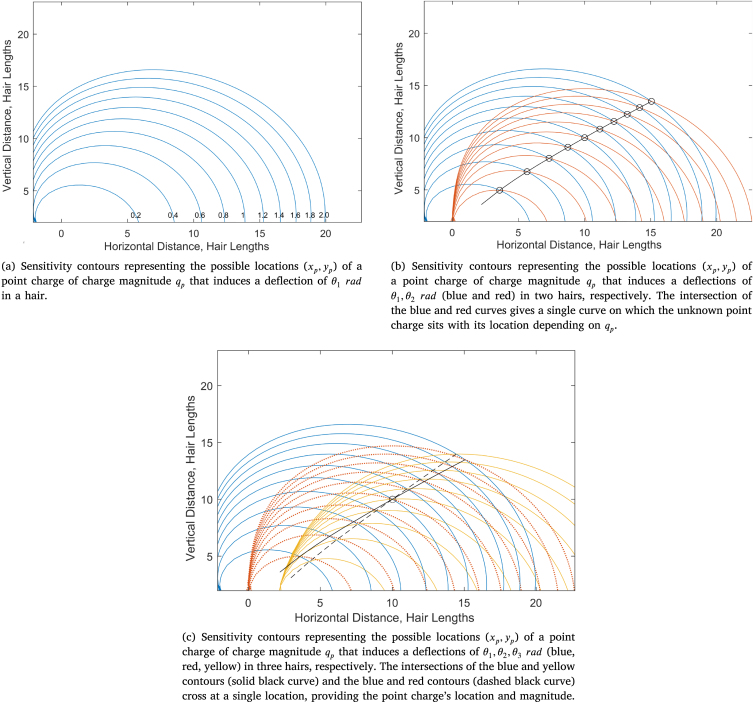
Relation between sensitivity contours for a three-hair system and geometry of charge detection.

For each hair, a set of contours are calculated that indicate the possible locations $\left(x_{p},y_{p}\right)$ of a point charge of a given magnitude ($q_{p}$) such that the hair deflects by $\theta_{h}\;\mathrm{rad}$ (as developed and presented in Palmer et al. (2022)) shown in Fig. 2. Considering a single hair, Fig. 2(a) shows ten contours for the hair on the left-hand side of the three. Each curve is produced by different charge magnitudes $q_{p}=0.2,\;0.4,\;...,\;2$. In this instance, considering all values of $q_{p}$ will produce contours that fill the domain.

Next, consider the contours of two hairs, say the left-hand and central hairs of the three. For each value of $q_{p}$ a point of intersection between the respective contours is found. Thus, the deflection of two hairs reduces the possible locations of a point charge from anywhere within the domain to a single continuous curve parameterised by $q_{p}$ (see Fig. 2(b) for an example of this).

Finally, from the deflection of three hairs, the above procedure is repeated pairwise to obtain three curves of the possible point charge locations (each parameterised by $q_{p}$). Considering two such curves (say, for hairs 1 and 2, and hairs 1 and 3), three scenarios are possible:

1. The two curves do not overlap. There is no feasible location for the point charge for any value of $q_{p}$ that would produce the known hair deflections $\theta_{1},\theta_{2}$ and $\theta_{3}$.
2. The curves overlap once and indicate the location of the point charge and the value of $q_{p}$.
3. The curves overlap twice. There are two possible locations and charge values for the point charge. In this case, the third curve will overlap with one of these points (if feasible) and determine the location of the point charge and the value of $q_{p}$.

Thus, for a single point charge and three hairs, the location of the point charge and its magnitude $q_{p}$ can be determined, illustrated by Fig. 2(c)

Having shown the conceptual possibility for the location and charge determination of a single point charge using an array of three hairs, we now generalise to the scenario of multiple point charges.

#### 3.1.2 Numerical example

Let us first consider a single point charge and an array of three hairs as in Section 3.1. With three hairs, the system of equations is fully determined (three equations for three unknowns).

**Fig. 3:**
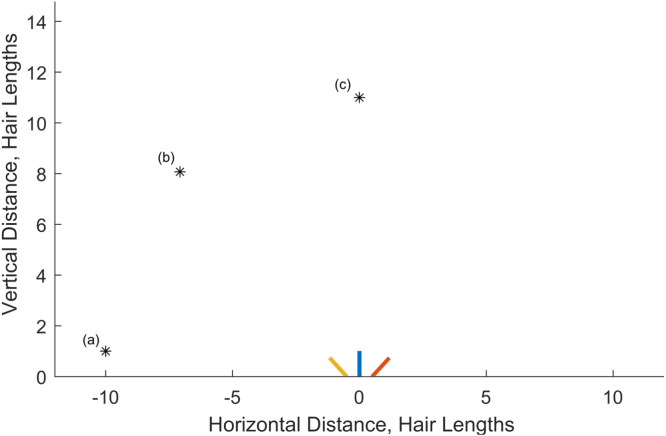
Diagram of the charge positions considered in the single charge detection analysis in Section 3.1.2.

To illustrate the detection ability of a hair array, we present three cases in which a single point charge is located in a different place around that array:

**(a)** To the left of the array and level with the resting position of the hair tips: $\left(x_{p},y_{p}\right)=\left(-10,1\right)$,
**(b)** Diagonally above the hair array: $\left(x_{p},y_{p}\right)=\left(-\sqrt{50},\sqrt{50}+1\right)$, and
**(c)** Directly above the array centre: $\left(x_{p},y_{p}\right)=\left(0,11\right)$.

as shown in Fig. 3. In each case, $q_{p}=1$ and the point charge is ten hair lengths away from the resting position of the central hair tip.

We randomly generate initial conditions for $\left(x_{p},y_{p}\right)$ and $q_{p}$ to solve Eqs. (3.1). For the presented cases we used:

$$\text{(1a)}\;q_{p,0}=3.47,\;x_{p,0}=2.22,\;y_{p,0}=-8.82,$$

$$\text{(1b)}\;q_{p,0}=-1.93,\;x_{p,0}=-24.44,\;y_{p,0}=-46.37,$$

$$\text{(1c)}\;q_{p,0}=6.21,\;x_{p,0}=30.45,\;y_{p,0}=5.14.$$

Notably, these values are far from the correct solutions.

For each case outlined above, Table 1 presents the accuracy of the point charge location and magnitude estimation. These are given by:

(3.2) $$\text{acc}\left(x_{p}\right)=\|x_{p}-{\overset{¯}{x}}_{p}\|,\;\text{acc}\left(q_{p}\right)=\frac{\left\|q_{p}-{\overset{¯}{q}}_{p}\right\|}{q_{p}},$$

where $x_{p}$ and $q_{p}$ are the actual values, and ${\overset{¯}{x}}_{p}$ and ${\overset{¯}{q}}_{p}$ are the computed values. Notably, the metrics for measuring the estimated location and magnitude accuracy (here and throughout) are non-dimensional. Table 1 shows that the estimated values are very close to the actual values. For example, the location metrics are all of magnitude 10^−6^ hair lengths due to the non-dimensionalisation. Thus, a 1 mm long arthropod hair predicts a point charge within $10^{-6}\;\mathrm{mm}$ of the actual location. The non-dimensionalisation is informative since it ensures that the results hold across different scales. Overall, both measures indicate the accuracy with which an array of three hairs detects a single point charge in different parts of the domain when all the hair parameters are known.

**Table 1:** Detecting one point charge: accuracy of location and charge strength estimation for a system of three hairs for three locations of the point charge: (1a) left of the hair array, (1b) above and left of hair array, (1c) directly above the hair array.

Cases	Metrics	
	$\text{acc}\left(x_{p}\right)$	$\text{acc}\left(q_{p}\right)$
(1a)	$5.1\times 10^{-6}$	$1.0\times 10^{-7}$
(1b)	$5.6\times 10^{-6}$	$9.9\times 10^{-8}$
(1c)	$9.6\times 10^{-6}$	$1.7\times 10^{-7}$

### 3.2 Detection of multiple point charges

**Fig. 4:**
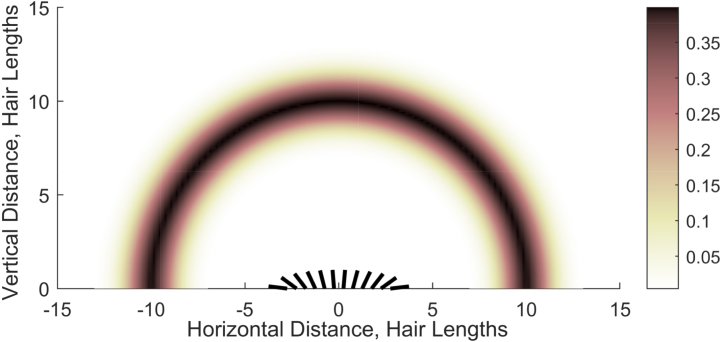
Diagram of the potential charge positions considered in the multiple charge detection analysis in Section 3.2. The point charge location is randomly assigned with its radius from the hair array being normally distributed ($r_{p}∼N\left(10,2^{2}\right)$) and its angular position uniformly distributed ($\phi_{p}∼U\left(-\pi /2,\pi /2\right)$). Thus, the colours indicate the probability that a point charge is at a given radius.

To further explore location detection through arrays of hairs, now consider the case of multiple unknown point charges. The aim is to examine how an array of 12 hairs can detect up to four unknown point charges with the number of point charges and their locations and magnitudes unknown. To this end, the point charge locations are assigned randomly within an arc about the hair array (illustrated in Fig. 4). The radial distance of each point charge from the origin is normally distributed such that $r_{p}∼N\left(10,2^{2}\right)$, whilst its angular position is uniformly distributed with $\phi_{p}∼U\left(-\pi /2,\pi /2\right)$. Therefore, in Fig. 4 the coloured arcs indicate the probability that a point charge is at a given radius. Furthermore, the magnitude of each point charge is normally distributed (and may differ from each other) such that: $q_{p}∼N\left(1,0.1^{2}\right)$.

For an initial condition, in each case we assume that four point charges of magnitude $q_{p}=1$ are equally distributed throughout the domain such that: $\left(r_{p},\;\phi_{p}\right)=\left(8,-\pi /2+p\pi /5\right)$, p=1,2,3,4. Notably, the choice of initial condition affects the accuracy of the presented analysis; thus, we chose these positions to give a broad coverage of the domain.

To analyse this setup, we produce 100 samples each of (a) one charge, (b) two charges, (c) three charges, and (d) four charges, and use three metrics. Firstly, we consider how accurately a system of twelve hairs can detect the number of charges that make up the external electrical field. Secondly, we calculate the Euclidean distance between the centre of charge for the actual electrical field and the observer’s reconstruction:

(3.3) $$x_{c}=\frac{\overset{P}{\underset{p}{\sum }}q_{p}x_{p}}{\overset{P}{\underset{p}{\sum }}q_{p}},\;y_{c}=\frac{\overset{P}{\underset{p}{\sum }}q_{p}y_{p}}{\overset{P}{\underset{p}{\sum }}q_{p}},$$

(3.4) $${\overset{¯}{x}}_{c}=\frac{\overset{\overset{¯}{P}}{\underset{p}{\sum }}{\overset{¯}{q}}_{p}{\overset{¯}{x}}_{p}}{\overset{\overset{¯}{P}}{\underset{p}{\sum }}{\overset{¯}{q}}_{p}},\;{\overset{¯}{y}}_{c}=\frac{\overset{\overset{¯}{P}}{\underset{p}{\sum }}{\overset{¯}{q}}_{p}{\overset{¯}{y}}_{p}}{\overset{\overset{¯}{P}}{\underset{p}{\sum }}{\overset{¯}{q}}_{p}},$$

(3.5) $$\text{acc}\left(x_{c}\right)=\|x_{c}-{\overset{¯}{x}}_{c}\|,$$

where δ=0.1, $\left(x_{p},y_{p}\right)$, $q_{p}$ and P are the actual values, and $\left({\overset{¯}{x}}_{p},{\overset{¯}{y}}_{p}\right)$, ${\overset{¯}{q}}_{p}$ and $\overset{¯}{P}$ are the computed values. This metric indicates whether the hair array has captured the overall charge in the system at the correct location. Furthermore, this metric enables the comparison of the actual electrical field to cases where the estimated number of charges does not equal the actual number. Thirdly, we calculate the proportional accuracy of the total estimated charge compared to the actual total charge:

(3.6) $$\text{acc}\left(\overset{P}{\underset{p}{\sum }}q_{p}\right)=\frac{\left\|\overset{P}{\underset{p}{\sum }}q_{p}-\overset{\overset{¯}{P}}{\underset{p}{\sum }}{\overset{¯}{q}}_{p}\right\|}{\overset{P}{\underset{p}{\sum }}q_{p}}.$$

Together with the centre of charge, these metrics indicate whether inaccuracies in the predicted charges are due to the estimated location or magnitude of charge.

**Fig. 5:**
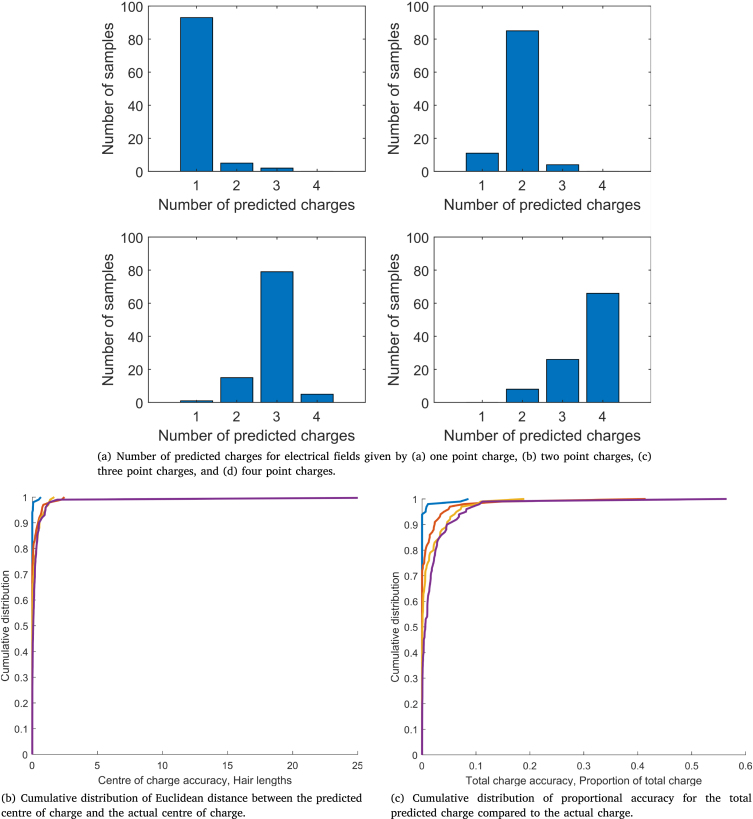
Metrics of the N unknown point charges analysis.

Fig. 5(a) presents the number of predicted point charges for each case. Overall, the accuracy of the predicted number of charges diminishes as the number of actual charges increases. There are two reasons for this. Firstly, when two or more charges are near each other, it is more difficult to predict their locations and magnitudes. For the cases of three and four point charges, there is a greater likelihood that the generated charges are close and therefore harder to distinguish. Thus, fewer point charges may be predicted due to their proximity, the quality of the initial condition and the distance from the hair array. Secondly, the system is over-determined in the single, two and three point charge cases since only three, six and nine hairs are required to locate the charge/charges, respectively.

To understand these results further, Fig. 5(b) shows the cumulative distribution of accuracy for the estimated centre of the charge. As before, the accuracy decreases with increasing numbers of actual charges. For the one point charge system the analysis is very accurate with 98% of cases having $\text{acc}\left(x_{c},y_{c}\right)\leq 0.1$. For two point charges, 82% of cases have $\text{acc}\left(x_{c},y_{c}\right)\leq 0.1$, whilst the three and four point charges are 70% and 55% accurate at the same level. Furthermore, larger inaccuracy can occur with more point charges. Significantly, for all four scenarios (1–4 point charges in space), at least 95% of the samples are accurate within one hair length. Overall, an array of twelve hairs can predict the structure of electrical fields consisting of four or fewer point charges within a hair length’s accuracy.

Finally, Fig. 5(c) presents the cumulative distribution of total charge accuracy. Similar trends are seen with the accuracy reducing when more point charges make up the actual electrical field. Yet, the results remain reasonably accurate for each scenario, with the majority of cases falling within 10% accuracy (98% of cases for two, three and four point charges, and 100% for one point charge).

**Fig. 6:**
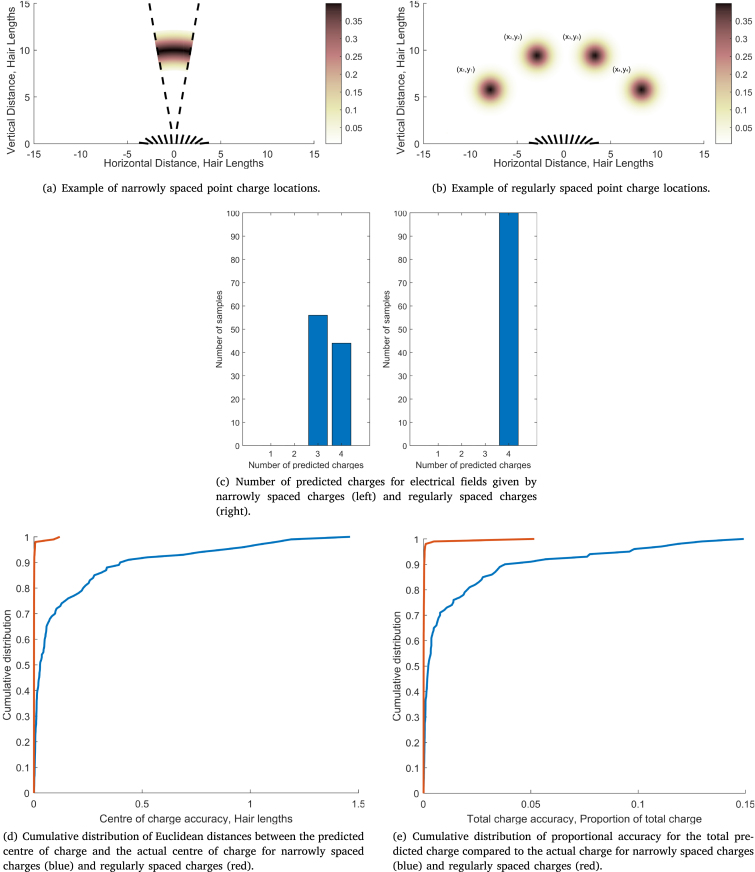
Comparison of metrics for 4 narrowly spaced charges and 4 regularly spaced charges.

The same metrics are applied to two more scenarios to assess how the proximity between charges affects location detection accuracy, shown in Fig. 6. The first scenario considers four narrowly spaced point charges, see Fig. 6(a). The locations of the point charges are determined using the same normally distributed radius as before; however, the angular position is now uniformly distributed over a narrower range, $\phi_{p}∼U\left(-\pi /18,\pi /18\right)$. A new initial condition for the charge locations is used with $r_{p}=8$ for p=1,2,3,4 and $\phi_{1}=-2\pi /18,\phi_{2}=-\pi /18,\phi_{3}=\pi /18,\phi_{4}=2\pi /18$.

The second scenario considers four regularly spaced point charges, shown in Fig. 6(b). Their locations distribute around equally spaced centres:

$$\left(x_{p,c},\;y_{p,c}\right)=10\;\sin\left(-\pi /2+p\pi /5\right),\;10\;\cos\left(-\pi /2+p\pi /5\right),\;p=1,2,3,4,$$

by a normally distributed radial distance $r_{p}∼N\left(0,1\right)$ and a uniformly distributed angular position $\phi_{p}∼U\left(-\pi ,\pi \right)$.

From Fig. 6(c), the number of narrowly spaced charges is harder to discern with the electrical field predicted to consist of three point charges in most cases, compared to the 100% accuracy for regularly spaced charges. Furthermore, Fig. 6, Fig. 6 respectively show the cumulative distributions for centre of charge and total predicted charge accuracy. The regularly spaced charges are (red curves) predicted with greater accuracy according to both metrics; however, the predicted centre of charge for narrowly spaced charges is still within a hair length of the actual for most cases.

These results indicate the difficulty in predicting the precise locations and magnitudes of narrowly spaced charges compared to regularly spaced charges from a static system of multiple hairs. Moreover, we expect the largest inaccuracy to occur when point charges align with the centre of the hair array. In such cases, it is more likely that the hair array will predict fewer charges depending on the distance between them. This highlights a geometrical issue with making a single observation from a single hair array. In reality, however, arthropods endowed with electroreceptor hairs could make a series of discrete or continuous observations to overcome this limitation by moving in an orthogonal direction. We now turn our attention to the case of distributed observations with more unknowns.

## 4 Characterising an electrical field using unknown hair parameters

Consider the situation in which an observer takes multiple observations of an electrical field. However, now the hair parameters are also unknown. Thus, we seek to show how arthropods may learn the mechanical and electrical properties of their hairs ($L_{h},S_{h},q_{h},K,\;h=1,2,\ldots ,H$) and the characteristics of external point charges ($x_{p},y_{p},q_{p},\;p=1,2,\ldots ,P$) through a series of observations.

For each observation, the arthropod moves freely around the domain translating its position by some vector, $\left(x_{k},y_{k},0\right)$, and rotating freely by some angle, $\phi_{k}$. As a result, at some time $t_{k}$, the hair base locations become:

(4.1) $$x_{0,h}\left(t_{k}\right)=\cos\left(\phi_{k}\right)x_{0,h}\left(0\right)+\sin\left(\phi_{k}\right)y_{0,h}\left(0\right)+x_{k},$$

$$x_{h}\left(t_{k}\right)=x_{0,h}\left(t_{k}\right)+L_{h}\sin\left(\theta_{h}\left(t_{k}\right)+\phi_{k}\right),$$

$$y_{0,h}\left(t_{k}\right)=-\sin\left(\phi_{k}\right)x_{0,h}\left(0\right)+\cos\left(\phi_{k}\right)y_{0,h}\left(0\right)+y_{k},$$

$$y_{h}\left(t_{k}\right)=y_{0,h}\left(t_{k}\right)+L_{h}\cos\left(\theta_{h}\left(t_{k}\right)+\phi_{k}\right).$$

Each observation satisfies H equations of the form (2.8) (with $A^{\left(h\right)}$ now containing several unknowns), so GH equations are obtained for G observations. For the solution to be unique, we assume that the distribution of the hair base locations and the observer’s location and orientation are known relative to some origin and that the hair deflections are known for each hair at each observation. Hence there are three unknowns for each point charge: the charge $q_{p}$, and location $\left(x_{p},y_{p}\right)$, and three unknown parameters for each hair: $S_{h},L_{h},q_{h}$, and a further unknown in the non-dimensional variable K. Thus, in total there are 3P+3H+1 unknowns. Overall,

(4.2) G≥⌈3P/H+3+1/H⌉,

observations are required to resolve the system.

We now show the feasibility of calculating the hair parameters and the point charge values through a series of observations.

**Fig. 7:**
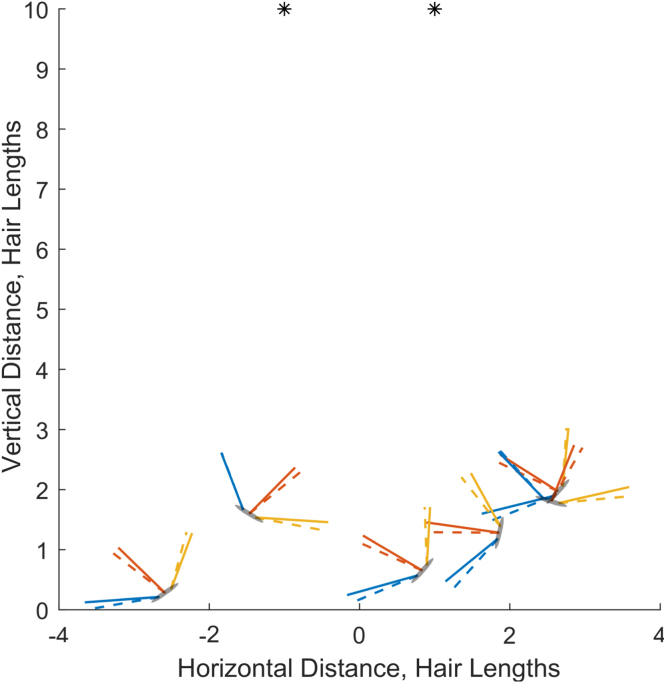
Example of six observations for parameter training. The black stars are two point charges, whilst the observer (grey ellipse) has three hairs (blue, red, yellow). Solid lines: hair positions at rest. Dashed lines: deflected hair positions.

**Table 2:** Training observations: mean, maximum and minimum accuracy in point charge location, hair length, $Kq_{h}q_{p}/S_{h}$ and $q_{p}/q_{h}$ for a system of three hairs taking 6 observations in a two point charge electrical field. The mean, maximum and minimum values are computed over all h=1,2,3 and p=1,2.

Stats	Metrics			
	$\text{acc}\left(x_{p}\right)$	$\text{acc}\left(L_{h}\right)$	$\text{acc}\left(K_{h,p}\right)$	$\text{acc}\left(Q_{h,p}\right)$
Mean	$9.6\times 10^{-4}$	$2.9\times 10^{-6}$	$9.9\times 10^{-4}$	$9.8\times 10^{-4}$
Max	$2.2\times 10^{-2}$	$2.1\times 10^{-4}$	$2.2\times 10^{-2}$	$2.2\times 10^{-2}$
Min	$3.6\times 10^{-7}$	$6.2\times 10^{-10}$	$2.8\times 10^{-7}$	$1.9\times 10^{-7}$

First, consider the external electrical field. For this example, the electrical field (unknown to the observer) consists of two point charges such that:

$$\left(x_{1},y_{1},q_{1}\right)=\left(-1,10,1\right),\;\left(x_{2},y_{2},q_{2}\right)=\left(1,10,1\right).$$

Secondly, consider the observer. Suppose it uses an array of 3 hairs (blue, red, yellow lines in Fig. 7) to detect the external electrical field. From Eq. (4.2) this three-hair system needs to make six observations to resolve the field presented in this example (point charges depicted by two asterisks in Fig. 7). For each observation, the location and orientation of the observer vary but are known and sampled from continuous uniform distributions:

$$x_{k}∼U\left(-3,\;3\right),\;y_{k}∼U\left(0,\;3\right),\;\phi_{k}∼U\left(-\pi /2,\;\pi /2\right)$$

Meanwhile, the hair parameters $L_{h},S_{h},q_{h}$ and K are unknown to the observer and randomly assigned from Gaussian distributions with mean μ=1 and standard deviation σ=0.01 for h=1,2,3. These remain fixed across the six observations (yet vary between samples) to compare the responses of arrays with dissimilar hairs. The small standard deviation ensures that the equilibrium positions of the hairs are not too extreme yet makes the hairs quantifiably different. An example of a single sample of 6 observations is shown in Fig. 7.

We produce 100 samples to assess the accuracy in determining the system parameters. In Table 2, the accuracy of point charge location and hair length estimation are given by:

(4.3) $$\text{acc}\left(x_{p}\right)=\|x_{p}-{\overset{¯}{x}}_{p}\|,$$

(4.4) $$\text{acc}\left(L_{h}\right)=\frac{\left\|L_{h}-{\overset{¯}{L}}_{h}\right\|}{L_{h}},$$

where $x_{p}$ and $L_{h}$ are actual values, and ${\overset{¯}{x}}_{p}$ and ${\overset{¯}{L}}_{h}$ are computed values. Regarding $q_{p},q_{h},S_{h}$ and K, from (2.8) it can be seen that $Kq_{h}L_{h}/S_{h}$ acts as a scaling parameter within the system. Thus, the values of each individual parameter cannot, and need not, be determined; however, the following two values are calculable:

$$K_{h,p}≔K\frac{q_{h}q_{p}}{S_{h}},\;Q_{h,p}≔q_{p}/q_{h}.$$

for h=1,2,3 and p=1,2, which suffices to solve the system. Together, we may also find $K\frac{q_{h}q_{i}}{S_{h}},\;i,h=1,2,\ldots ,H$. We, therefore, calculate the accuracy of these as follows, which are also reported in Table 2:

(4.5) $$\text{acc}\left(K_{h,p}\right)=\frac{\left\|K_{h,p}-{\overset{¯}{K}}_{h,p}\right\|}{K_{h,p}},$$

(4.6) $$\text{acc}\left(Q_{h,p}\right)=\frac{\left\|Q_{h,p}-{\overset{¯}{Q}}_{h,p}\right\|}{Q_{h,p}},$$

Overall, the results presented in Table 2 for 100 samples show that the process of learning parameters through several observations and hair deflections is very accurate for each metric. Thus, an observer can learn the electrical and mechanical properties of its hairs and the structural and electrical characteristics of the external electrical fields through a series of observations, using only knowledge of its location and orientation in the domain.

## 5 Classical navigation via electroreception

To further illustrate the potential of electroreception for location detection, we now consider the case of “classical navigation” where the location of the observer is unknown. In essence, this method is similar to stellar navigation in that the external field is assumed known and fixed such that the observer may use these points of reference to determine its location and orientation. To this end, we assume that the observer’s location and orientation are unknown, but all of their parameters that relate to hairs are known, likewise, the point charge locations and magnitudes are known.

**Fig. 8:**
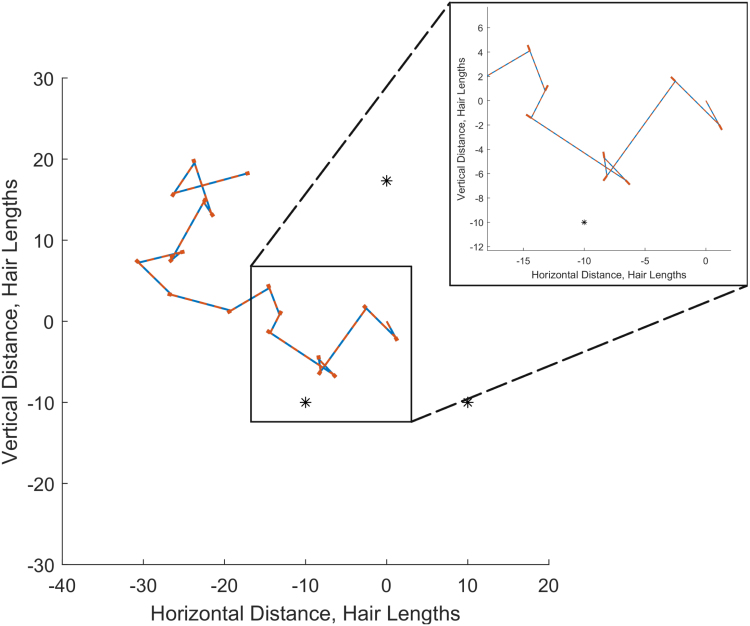
Classical navigation in an electrical fields consisting of three point charges (black stars) of magnitude $q_{p}=10$. The observer moves according to a random walk and can accurately track their location and orientation in space through the deflections of hairs alone. Red lines indicate the true location and orientation. Blue dashed lines indicate the predicted location and orientation.

We now solve (3.1) using the definitions of multiple observations from (4.1); however, the unknowns are $x_{k},y_{k},\phi_{k},\;k=1,2,\ldots ,G$. Since there are three unknowns for each observation, only three point charges are required to fully determine the system for each observation. Thus, for this example, the external field consists of three point charge organised as an equilateral triangle about the origin as seen in Fig. 8.

The observer begins at the origin in a known orientation. We model its motion using a random walk, hence this analysis does not involve any decision-making. Rather, as the observer moves randomly about the domain, the question is are they able to determine their location and orientation solely through the deflections of their hairs?

An example of this classical navigation is shown in Fig. 8. The red line segments are the true path that the observer travels, with the thick red line segments indicating the true orientation of the observer at each time step. The predicted path is plotted in dashed blue and matches the true path very closely. Indeed, in each case, the accuracy in both the observer’s location or orientation at each time step is within 10^−8^ hair lengths and 10^−5^ radians. Importantly, the path taken shows that this process can be accurate both inside and outside the triangular electrical field of the point charges. Overall, this analysis demonstrates the possibility that an observer can accurately determine its location and orientation within a known electrical field based solely on its hair deflections.

A note on the computational method, the solution scheme cannot handle discontinuities. Thus, for each step taken, the rotation and translation are discretised and solved over several steps. For example, if the observer rotated from 0 radians to π radians, to solve the equations the observer’s orientation must be incrementally updated at intervals of at least π/10 magnitude. Furthermore, to calculate the next step the previously estimated location is used as the initial condition.

## 6 Discussion

The presented theoretical analysis reveals the possibility that arthropods can detect and locate sources of electrical information solely through electrostatic interactions with their sensory hairs. Each study indicates a previously underappreciated sensory function of electroreception. In particular, from the deflection of multiple sensory hairs, it is possible to: (1) discern the location and magnitude of multiple point charges using a single static array, (2) learn the electrical and mechanical properties of a detector and the characteristics of an electrical field through a series of observations, and (3) determine an observer’s location and orientation within a fixed and known electrical field. Importantly, this analysis was conducted using biologically relevant and physically feasible parameters.

Several aspects of the modelling presented above require further comments on how such electrosensory capabilities may translate into a real-world setting. Firstly, the theory developed above depends on several discrete observations, often from randomly spaced locations. In reality, as an arthropod moves within a domain, it may observe the environment through deflections of its electromechanically sensitive hairs at a higher resolution than modelled here in a discrete or continuous manner. The ability to detect electrical fields is poised to be enhanced by a high rate of information acquisition through continuously moving filiform hairs, enabling a sufficiently accurate representation of the electric environment. The notion of continuously moving hairs may thus have thought-provoking and far-reaching consequences for how an arthropod moves and makes decisions, e.g. the paths it takes to gain heuristically sufficient information to make informed behavioural and navigational decisions. Crucially, for such hair sensing systems, the rate of information acquisition – a form of sampling rate – is not well defined, and therefore the notion of a truly continuous detection or perception remains questionable. The question of continuity, whilst valid for other sensory modalities as well, is intriguing and relevant here since electrostatic fields are continuous by nature. Thus, whilst filiform hairs are likely to be under a continuous electrostatic interaction, their mechanosensory neurones may transduce mechanical inputs into a discretised neural code that is limited in its frequency representation. In effect, spatial and temporal continuity in sensory perception is often seen as an illusion generated by neural processing filling detection gaps and is not necessarily related to the true continuous or discrete nature of the incoming stimulus.

Secondly, arthropods, and especially pollinators, are typically hairy. Roquer-Beni and colleagues systematically and carefully classified the density of hairs covering 109 pollinating insect species (Roquer-Beni et al., 2020). Remarkably, great diversity emerged from this analysis, even amongst closely related species, with hair density and organisation varying over the cuticular surface area of a single insect, depending on the body part considered. Overall, they found that hair coverage for several pollinator species to be in the range of 100–1000 hairs per ${\mathrm{mm}}^{2}$. Even though not every hair observed is mechano-sensory (or electromechanosensory), our analysis, based on a few hairs only, is likely to underestimate the putative sensory capacity in real-world arthropods.

Thirdly, the electrical fields presented throughout are static and arise from several discrete point charges. Natural electrical fields, however, derive from continuous charge densities that may fluctuate in space and time. Nevertheless, the results and predictions presented here will translate to real-world continuity for four main reasons. (i) Natural electrical fields will have significant diversity in geometry. Hence, those produced in the environment by flowers, for example, will present electrical peaks in regions with sharp angles (such as high curvature geometry) and when charge mobility is significant. Moreover, it is expected that large local electric fields can result from electrical induction and polarisation in these floral systems. In these scenarios, discrete point charges can appropriately represent such differences and variations in electric fields. (ii) In general, for a sufficiently large number of point charges, a discretised electrical field provides an accurate and reasonable approximation of continuous electrical fields. (iii) The hair arrays consist of several distributed and discrete sensors that convey geometrical information about an electrical field. Therefore, the reproduced image is inherently discrete, as with other sensory systems. Indeed, the discretisation of the electrical field is a restriction on how the field is viewed or detected, rather than being representative of its construction. As a result, the methods presented hold for continuously generated fields with the hair deflections producing a discretised approximation of such fields. Assuming that the observer “views” a continuously generated electrical field as being formed of a collection of discrete points (with some restriction on resolution due to the number of hairs and observations), the process outlined above will provide a reasonable approximation. (iv) Arthropods are distinctly hairier than considered here. Furthermore, their sensory hairs distribute over different surface geometries and appendages, with arthropods able to make multiple observations in time. These points reduce the limitations of the discretised representation of the electrical fields since they can be portrayed with a higher resolution than considered here.

Fourthly, considering spatio-temporal effects further, environmental electrical fields are known to vary with time due to external influences (e.g. weather gradients, plants, other animals and arthropods) and spatial charging dynamics (e.g. triboelectric versus inductive charging mechanisms between observer and environment). With these considerations, the dynamic terms in (2.3) for angular acceleration and angular velocity of the hairs will be influential. If these are measurable by the hair systems, the current analysis can be readily extended to capture time-varying dynamics. Interestingly, should the angular accelerations and velocities remain unknown, it is still theoretically possible, with enough hairs, for an organism to gather sufficient information to determine the electrical field (indicated by Section 4). In effect, the quasi-static system constitutes a good approximation when the contributions of these dynamic hair terms are small or when the values of I and R are small.

Fifthly, under dynamic conditions, other influences such as aero-acoustic sensing arise. Thus, the mutual effects of multiple stimuli on hair movement require careful consideration. This multi-modality is the basis of future work. As discussed in both Palmer et al. (2021) and Palmer et al. (2022), when both the aerodynamic and electrostatic hair responses are considered, variation in the morphology of the hair array (such as hair lengths and spacing) becomes increasingly influential. In particular, the synergistic relationships between hairs through electrostatic coupling (Palmer et al., 2022) and viscous-mediated coupling (Bathellier et al., 2005, Cummins et al., 2007) need to be quantified to assess how each affects the ability to perform tasks such as localisation and acoustic signal discrimination. If the time and length scales of electroreception and electrical stimuli are distinct from those of aero-acoustic sensing, the response of a hair array to multiple stimuli could be decoupled. Hence, multiple hairs distributed over an arthropod may constitute a dispersed collective sensor that enables several distinct and simultaneous stimuli to be independently discernible. This enticing and disruptive hypothesis requires further examination.

### A comment on bimodal sensing.

Naturally occurring electrical fields and stimuli are ubiquitous within nature. Their prevalence is a consequence of the electrical properties (e.g., conductive, inductive and triboelectric charging), geometry and natural composition of biological materials, and their interactions with and within the environment. However, aero-acoustic stimuli are not universally present in nature in the same manner as electrical fields (e.g. a flower will readily emit an electrical field but most likely not an aero-acoustic signature). Moreover, the physical description of aero-acoustic stimuli is distinct from electrical stimuli; thus, each stimuli has a different effect on filiform hairs. These differences enable the processes of electrical localisation outlined in this article. Notably, location detection via acoustic hair sensing is possible in the context of predator–prey or mating interactions, for example (e.g., detecting the wing beat of another arthropod and its location). Yet, here we expect electrostatic interactions can convey further and perhaps richer information to the observer. For example, in the process of proximity detection. Previously, cricket flow sensing hairs have been shown to act as proximity detectors for predators (Casas and Steinmann, 2014, Steinmann and Casas, 2017). Considering the additional sensory capabilities presented in the current manuscript, electroreception may further strengthen such processes. The possibility of proximity detection in electrostatic sensory hair systems has been discussed further in a previous paper (Palmer et al., 2022). Currently, we are pursuing several experiments to test the hypotheses and theory presented in this paper and our previous work in relation to the new sensory capabilities of electroreception and inter-hair dynamics.

Furthermore, the bimodality of hair sensing in nature where abundant sources of continuous aero-acoustic and electrical stimuli arise is a complex phenomenon and highlights a significant outstanding question: can aero-acoustic and electrical stimuli be distinguished by one (or few) sensory hair endowed with a single (or few) mechanosensory neuron? And, if so, what processes enable this? Currently, we have two hypotheses.

Firstly, bimodal signal processing may be possible through the mechanics and dynamics of sensory hair arrays. The combination of multiple hairs deflecting may discriminate signals through emergent dynamics that depend on the physical natures of the different signals. We anticipate that hair motions and inter-hair influences will vary depending on modality due to the differences in the underpinning physics of aero-acoustic signals (e.g. frequency based signals, particle velocity representations, directionality, impulses vs acoustic) and electrical signals (e.g. spatial and geometrical characteristics of electrical fields described by Poisson equations, charging dynamics such as polarisation). Furthermore, in electrostatics there is no biological equivalent of a sound wave (i.e. pure AC signals that switch polarity are not expected in nature, though at proximity an arthropod’s wing beat frequency may resemble a sinusoidally modulated DC signal), as such the leading order time scale of electrostatic signals is expected to differ from aero-acoustic signals. The above points to each modality possessing distinct spatio-temporal characteristics that command different hair dynamics. Hence, they may be naturally decomposed, and thus differentiated, by the collective motion of a sufficient number of hairs.

Secondly, signal differentiation may occur neurologically. Sensory hairs are typically innervated with one or few neurons (1 to 4–5, depending on species) such that when a hair’s deflection exceeds a given threshold, its primary sensory neurone fires and encodes sensory information in one of two ways: as continuous deflection measurement (tonic coding) or action potential (phasic coding). Depending on the as-yet-discovered neurological process, the modality and strength of an incident signal may be distinguished at the stage of detection by the primary neurone through: amplitude-dependant temporal integration (a process known to take place in mechanoreceptive scolopidial units) or differential frequency tuning (whereby low frequency aero-acoustics are distinct from higher frequency and bandwidth electrical transients). Furthermore, mechanoreceptive hairs have been shown to be highly sensitive, with small deflections in the order of 0.001 rad and below able to elicit neurological responses (examples of experimentally verified sensitivity thresholds include: 0.0032rad∼0.2° for crickets (Shimozawa et al., 2003), 0.0016−0.016rad∼0.1−1° for the trichobothria of *Cupiennius salei* spiders (Barth and Höller, 1999) and 0.0007rad∼0.04° for Bumblebee (*Bombus terrestris*) hairs (Sutton et al., 2016)). Hence, by a single hair, small changes in a mechanical stimulus can be neurologically detected, with several hairs able to distinguish many differences and variations in both space and time. Indeed, information acquisition and discrimination via ensembles of spatially-distributed and differently tuned sensory hairs, will yield a range of neurological responses within their associated primary neurones, as previously shown (e.g., Steinmann and Casas, 2017, Theunissen et al., 1996), strengthening the possibility of modality discrimination. Experiments are being undertaken to quantify the timing and strength of action potential-based coding in both cricket cercal and Bumblebee cranial hairs.

### Empirical evidence and verification.

Currently, empirical evidence of electroreception demonstrates the behavioural implications of this sense, providing evidence for the detection of ambient weak electric field. This evidence stems from work on spiders (Morley and Robert, 2018), bumblebees (Clarke et al., 2013), honeybees (Greggers et al., 2013, Amador et al., 2017) and hoverflies (Khan et al., 2021). In each case, the primary candidate for the electrosensor is mechanosensory hairs (England and Robert, 2021). However, the biological sensory processes that electroreception may enable and the inter-hair interactions that arise remain largely unexplored. The analysis presented above is instructive in this manner and highlights ways forward. It demonstrates the significant role that electroreception may play in the sensory ecology and behaviour of arthropods, disrupting the existing theory and understanding whilst opening many avenues for future study.

To test the hypotheses of this paper experimentally the following require further investigation: (1) the spatio-temporal characteristics of natural e-fields, (2) the relative strengths of aero-acoustic stimuli to electrical stimuli in different contexts and scenarios, and (3) the role of polarisation and co-emergent electrical interactions in different ecological contexts, for example predator–prey interactions or pollinator-flower interactions. Regarding the relative strengths of different stimuli, this is a nuanced point that depends on the relevant biological context. Both modalities are expected to vary differently with space and time, thus we need to understand the correct time and space scales (and thus biological scenarios and the arthropod’s behaviour) in which different stimuli dominate or are comparable. However, accurately recreating ecologically relevant signals within a laboratory setting is difficult. As such, we propose that an allied approach between mathematical modelling and empirical investigation will be a powerful tool for answering the outstanding questions. Empirically, the relevant experimental methods continue to include micro-scanning Laser Doppler Vibrometry and neurophysiological methods applied to a wider range of well-chosen arthropod species. Furthermore, reproducing previously published aero-acoustic experiments with new electrical stimuli present would be a fruitful avenue for further empirical evidence.

A further complication in empirically assessing the role of sensory hairs in electroreception, and perhaps other modalities, is that not all arthropod hairs are innervated nor mechanoreceptive and thus the proximity of sensory hairs to one another and the interactions between them are difficult to quantify. For example, honeybees possess several types of hair that perform distinct tasks (e.g. pollen capture, thermal insulation, olfaction (Southwick, 1985, Clarke et al., 2017, Khan and Liu, 2022)). Hence our model requires further adaptation to reflect a multi-hair array that includes unarticulated hairs for example. Nonetheless, the interactions between sensory and non-sensory hairs may produce significant dynamics such that their configuration and physical properties play a crucial sensory role. For example, the presence of unarticulated hairs can significantly change the response of neighbouring hairs to aero-acoustic stimuli (Bathellier et al., 2005, Cummins et al., 2007), modulating their sensitivity. We expect similar results for electrostatic coupling (Palmer et al., 2022). Furthermore, other arthropods such as spiders and crickets can possess more regularly ordered sensory hairs (with different spacing) that may be more readily reflected by our model.

## 7 Conclusions

Each discovery in this article is a unique and powerful sensory capability that electroreception enables and conveys to an observer. Their uniqueness lies in the ability to discern information about an electrical environment solely through the mechanical deflections of sensory hairs.

Overall, the simplified scenarios presented here become very powerful when translated into the real world. Enticingly, when combined with an increased number of distributed sensors and continuity of observations (as noted above), the findings here open up the possibility that many arthropod species may be able to map their electrical environment in real-time. Hence, each point of the above discussion is a crucial avenue for future work.

Whilst filiform hairs are known to be sensitive to several stimuli, the functional significance of the electromechanical sensory modality is only beginning to be addressed, and its broader implications realised. Thus, an exciting, outstanding question is: how widespread and functional could such a sense be?

Alongside the evidence of electroreception within specific species listed throughout this paper, there is growing evidence of this sense within the broader diversity of terrestrial arthropods (England and Robert, 2021). If further evidence shows that this sense is more widespread amongst arthropods, it may be that most animal species possess this sense. The widespread phyletic presence of aerial electroreception would fundamentally change our understanding of sensory ecology, the ecology of information, species interactions, and, therefore, the structures and functions of natural ecosystems. In addition, such discoveries will increase the urgency with which we need to understand the potential direct and indirect effects of anthropogenic electricity on ecosystems (e.g. electrical powerlines, electromagnetic spectrum and its power, electrostatics and aerosols). This work is only the beginning of our exploration of the pervasiveness and interconnectedness of the natural electrical world and the activities of animals therein. From the interactions between flowers, pollinators, microbes, fungi, plants and local weather gradients, to the large scales of atmospheric processes that drive electrical fields, to the small-scale charging of hairs and their electric interactions, electrical processes occur throughout the world across vast length scales and the significance and importance of which is being shown to be greater than previously imagined. Therefore, more empirical and theoretical studies are required to understand the rich and complex sources of information that electrical fields provide.

## CRediT authorship contribution statement

**Ryan A. Palmer:** Conceptualization, Methodology, Formal analysis, Writing – original draft. **Isaac V. Chenchiah:** Conceptualization, Writing – review & editing, Supervision, Funding acquisition. **Daniel Robert:** Conceptualization, Writing – review & editing, Supervision, Funding acquisition.

## Declaration of Competing Interest

The authors declare that they have no known competing financial interests or personal relationships that could have appeared to influence the work reported in this paper.

## Data Availability

No data was used for the research described in the article.

## References

[b1] Amador G.J., Matherne M., Waller D., Mathews M., Gorb S.N., Hu D.L. (2017). Honey bee hairs and pollenkitt are essential for pollen capture and removal. Bioinspiration Biomim..

[b2] Barth F.G., Höller A. (1999). Dynamics of arthropod filiform hairs. V. The response of spider trichobothria to natural stimuli. Phil. Trans. R. Soc. of Lond. B.

[b3] Bathellier B., Barth F.G., Albert J.T., Humphrey J.A. (2005). Viscosity-mediated motion coupling between pairs of trichobothria on the leg of the spider *Cupiennius salei*. J. Comp. Physiol. A.

[b4] Casas J., Dangles O. (2010). Physical ecology of fluid flow sensing in arthropods. Annu. Rev. Entomol..

[b5] Casas J., Steinmann T. (2014). Predator-induced flow disturbances alert prey, from the onset of an attack. Phil. Trans. R. Soc. of Lond. B.

[b6] Casas J., Steinmann T., Dangles O. (2008). The aerodynamic signature of running spiders. PLoS One.

[b7] Clarke D., Morley E., Robert D. (2017). The bee, the flower, and the electric field: electric ecology and aerial electroreception. J. Comp. Physiol. A.

[b8] Clarke D., Whitney H., Sutton G., Robert D. (2013). Detection and learning of floral electric fields by bumblebees. Science.

[b9] Cocroft R.B., Rodríguez R.L. (2005). The behavioral ecology of insect vibrational communication. Bioscience.

[b10] Cummins B., Gedeon T., Klapper I., Cortez R. (2007). Interaction between arthropod filiform hairs in a fluid environment. J. Theor. Biol..

[b11] England S.J., Robert D. (2021). The ecology of electricity and electroreception. Biol. Rev..

[b12] Greggers U., Koch G., Schmidt V., Dürr A., Floriou-Servou A., Piepenbrock D., Göpfert M.C., Menzel R. (2013). Reception and learning of electric fields in bees. Phil. Trans. R. Soc. of Lond. B.

[b13] Hill P.S.M., Wessel A. (2016). Biotremology. Curr. Biol..

[b14] Humphrey J.A., Barth F.G. (2007). Medium flow-sensing hairs: biomechanics and models. Adv. Insect Physiol..

[b15] Khan S.A., Khan K.A., Kubik S., Ahmad S., Ghramh H.A., Ahmad A., Skalicky M., Naveed Z., Malik S., Khalofah A. (2021). Electric field detection as floral cue in hoverfly pollination. Sci. Rep..

[b16] Khan K.A., Liu T. (2022). Morphological structure and distribution of hairiness on different body parts of apis mellifera with an implication on pollination biology and a novel method to measure the hair length. Insects.

[b17] Koh K., Robert D. (2020). Bumblebee hairs as electric and air motion sensors: theoretical analysis of an isolated hair. J. R. Soc. Interface.

[b18] Kumagai T., Shimozawa T., Baba Y. (1998). The shape of wind-receptor hairs of cricket and cockroach. J. Comp. Physiol. A.

[b19] Liu F., Wang Y., Zhao Y., Liu M., Hou T., Han Z. (2022). Target-oriented passive localization techniques inspired by terrestrial arthropods: a review. J. Bionic Eng..

[b20] Morley E.L., Robert D. (2018). Electric fields elicit ballooning in spiders. Curr. Biol..

[b21] Palmer R.A., Chenchiah I.V., Robert D. (2021). Analysis of aerodynamic and electrostatic sensing in mechanoreceptor arthropod hairs. J. Theoret. Biol..

[b22] Palmer R.A., Chenchiah I.V., Robert D. (2022). The mechanics and interactions of electrically-sensitive mechanoreceptive hair arrays of arthropods. J. R. Soc. Interface.

[b23] Robert D. (2005). Sound Source Localization.

[b24] Roquer-Beni L., Rodrigo A., Arnan X., Klein A.-M., Fornoff F., Boreux V., Bosch J. (2020). A novel method to measure hairiness in bees and other insect pollinators. Ecol. Evol..

[b25] Shimozawa T., Kumagai T., Baba Y. (1998). Structural scaling and functional design of the cercal wind-receptor hairs of cricket. J. Comp. Physiol. A.

[b26] Shimozawa T., Murakami J., Kumagai T. (2003). Sensors and Sensing in Biology and Engineering.

[b27] Southwick E.E. (1985). Bee hair structure and the effect of hair on metabolism at low temperature. J. Apicult. Res..

[b28] Steinmann T., Casas J. (2017). The morphological heterogeneity of cricket flow-sensing hairs conveys the complex flow signature of predator attacks. J. R. Soc. Interface.

[b29] Sutton G.P., Clarke D., Morley E.L., Robert D. (2016). Mechanosensory hairs in bumblebees (*Bombus terrestris*) detect weak electric fields. Proc. Natl. Acad. Sci..

[b30] Tautz J. (1979). Reception of particle oscillation in a medium—an unorthodox sensory capacity. Naturwissenschaften.

[b31] Tautz J., Markl H. (1978). Caterpillars detect flying wasps by hairs sensitive to airborne vibration. Behav. Ecol. Sociobiol..

[b32] Theunissen F., Roddey J.C., Stufflebeam S., Clague H., Miller J.P. (1996). Information theoretic analysis of dynamical encoding by four identified primary sensory interneurons in the cricket cercal system. J. Neurophysiol..

[b33] Thurm U. (1965). Cold Spring Harbor Symposia on Quantitative Biology.

[b34] Thurm U. (1965). Cold Spring Harbor Symposia on Quantitative Biology.

[b35] Warrant E., Nilsson D.-E. (2006).

